# Effectiveness of preoxygenation by conventional face mask versus non-invasive ventilation in morbidly obese patients: measurable by the oxygen-reserve index?

**DOI:** 10.1007/s10877-022-00825-1

**Published:** 2022-02-15

**Authors:** Janina Bathe, Sadia Malik, Hans O. Pinnschmidt, Amelie Zitzmann, Christoph R. Behem, Constantin C. Trepte, Daniel A. Reuter

**Affiliations:** 1 Center of Anaesthesiology and Intensive Care Medicine, Hamburg-Eppendorf University Medical Center, Martinistr. 52, 20246 Hamburg, Germany; 2 Institute for Biostatistics, Hamburg-Eppendorf University Medical Center, Hamburg, Germany; 3 Department of Anaesthesiology and Intensive Care Medicine, Rostock University Medical Center, Rostock, Germany

**Keywords:** Oxygen reserve index (ORI), Bariatric patients, Preoxygenation, NIV-preoxygenation

## Abstract

Preoxygenation is a crucial manoeuvre for patients’ safety, particularly for morbidly obese patients due to their reduced pulmonary reserve and increased risk for difficult airway situations. The oxygen reserve index (ORI™) was recently introduced as a new parameter of multiple wavelength pulse oximetry and has been advocated to allow assessment of hyperoxia [quantified by the resulting arterial oxygen partial pressure (*P*aO_2_)]. This study investigates if ORI can be used to evaluate the impact of two different preoxygenation manoeuvres on the grade of hyperoxia. Two preoxygenation manoeuvres were sequentially evaluated in 41 morbidly obese patients: First, breathing 100% oxygen for 5 min via standard face mask. Second, after achieving a second baseline, 5 min of non-invasive ventilation (NIV) with 100% oxygen. The effect of preoxygenation on ORI compared to *P*aO_2_ was evaluated and whether differences in the two preoxygenation manoeuvres can be monitored by ORI. Overall correlation of *P*aO_2_ and ORI was significant (Spearman-Rho coefficient of correlation 0.818, p < 0.001). However, ORI could not differentiate between the two preoxygenation manoeuvres although the *P*aO_2_ values for NIV preoxygenation were significantly higher compared to standard preoxygenation (median 505 mmHg (M1) vs. 550 mmHg (M3); p < 0.0001). In contrast, ORI values did not differ significantly (median 0.39 (M1) vs. 0.38 (M3); p = 0.758). Absolute values of ORI cannot be used to assess effectiveness of a preoxygenation procedure in bariatric patients, mainly because its range of discrimination is considerably lower than the high ranges of *P*aO_2_ attained by adequate preoxygenation. *Trial registration* German Clinical Trials Register: DRKS00025023 (retrospectively registered on April 16th, 2021).

## Introduction

Sufficient preoxygenation is a crucial step prior to induction of anaesthesia to provide safety for patients [1, 2]. This induction of hyperoxemia opens up a safety margin during airway management to prevent life-threatening hypoxemia. In particular patients with reduced pulmonary function and morbidly obese patients are at increased risk for such complications [3]. Here, preoxygenation by non-invasive ventilation (NIV) before anaesthesia is described to be associated with a significant improvement in oxygenation before tracheal intubation compared with standard preoxygenation [4, 5]. Using NIV for preoxygenation increases alveolar ventilation, which provides additional oxygen (O_2_) and allows more carbon dioxide (CO_2_) removal from alveolar gas [6]. Apart from the oxygen supply the partial arterial pressure of O_2_ (*P*aO_2_) also depends on the matching of ventilation and perfusion. Pulmonary vascular resistance decreases by high values of O_2_, low partial arterial pressure of CO_2_ (*P*aCO_2_), and increased pulmonary venous pressure [7]. NIV influences these parameters so ventilation/perfusion ratio should also be better [6]. Whatever way for preoxygenation is used—knowing that the provided preoxygenation procedure was effective would be a big step for patient safety.

Oxygen is mainly transported being bonded to haemoglobin molecules in the blood. At about 100 mmHg the curve is almost horizontal, meaning the haemoglobin is fully saturated and cannot bind any more oxygen molecules. At normal oxygen levels in the air and normal pressure of the atmosphere only a very small fraction of oxygen is physically dissolved in the blood (at a *P*aO_2_ of 100 mmHg only 3 ml per 1 L blood). But being directly dependent on the partial arterial pressure of oxygen, this fraction rises during a preoxygenation process [7]. Thus, effectiveness of preoxygenation is directly dependent on the level of oxygen partial pressure in the arterial blood that is reached by the preoxygenation manoeuvre. Knowledge about the effectiveness of the preoxygenation procedure would be highly desirable. Measuring the *P*aO_2_ is only possible by taking a blood gas sample from an arterial line, which is an invasive manoeuvre, only punctiform and the results come with a certain delay. In daily routine, we are limited to the measurement of peripheral oxygen saturation by pulse oximetry and to defining minimum time frames of preoxygenation.

Recently, a new variable, the so-called oxygen reserve index (ORI™, Masimo Corp, Irvine, USA) was introduced. The ORI is generated by a multiple wavelength pulse co-oximeter that uses the altered background light absorption due to changes in venous oxygen saturation to calculate the ORI using a proprietary algorithm. The ORI is a relative index that quantifies hyperoxemia in a non-invasive manner with a unit-less scale between 0—no hyperoxemia, and 1—maximum measurable hyperoxemia. First data in ranges of moderate hyperoxemia beyond *P*aO_2_ 200 mmHg showed promising correlations between changes in ORI and *P*aO_2_ [9].

This study investigates if effectiveness of preoxygenation in morbidly obese patients can be assessed by ORI. Secondly, this study examines, if differences in the effectiveness of preoxygenation via face mask or NIV can be monitored by ORI.

## Methods

This study was performed at the Hamburg-Eppendorf University Hospital from May 2017 until May 2018. We studied patients scheduled for bariatric surgery such as gastric banding and sleeve gastrectomy.

The ethical review board of the local authorities (Ethikkommission der Ärztekammer Hamburg, Hamburg, Germany; Chairperson Prof. Dr. med. R. Stahl, PV5404) approved the study on 24th January 2017. Inclusion criteria were morbid obesity (body mass index > 35), age over 18 years, and written consent to participate in the study. Exclusion criteria were any pre-existing severe structural lung disease.

No patient received any pharmaceutical premedication prior to surgery. After arrival in the operating room, all patients received standard monitoring according to the local protocol for bariatric surgery. This includes 5-lead ECG, pulse oximetry, and invasive arterial blood pressure monitoring via the radial artery. Furthermore, we placed an ORI sensor (Radical-7, Touch Screen Software version V1451i; Root Software version V1470i; MS-5 DSP version V7B16; Masimo, Irvine, CA) on the patients’ ring or middle finger of the non-dominant hand. Possible nail polish was removed beforehand. To shield the sensor from light and reduce the risk of artefacts we covered the finger with the sensor with a double layer of gauze and tape. Patients were asked to keep the arm and finger steady in a relaxed position. All patients were in supine position with a slightly elevated upper body (10–15°).

### Study protocol

**Fig. 1:**
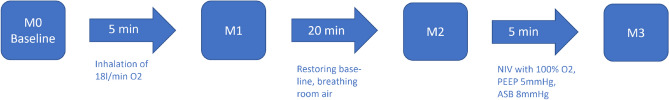
Study protocol. M, measuring point; min., minutes; O_2_, oxygen; NIV, non-invasive ventilation; PEEP, positive end-expiratory pressure; ASB, assisted spontaneous breathing mode. After taking baseline measurements the first preoxygenation manoeuvre was initiated by applying 100% oxygen for five minutes via a standard face mask. Then baseline was restored by breathing room air for 20 min before 5 min of NIV preoxygenation was started

The study protocol (Fig. 1) comprised basically of two consecutive preoxygenation manoeuvres. First, a baseline measurement (measuring point M0) was taken breathing room air. All vital parameters (heart rate (HR), mean arterial blood pressure (MAP), peripheral oxygen saturation (SpO_2_), and ORI) were measured. Additionally, an arterial blood gas analysis (BGA) was performed to assess arterial oxygen partial pressure (*P*aO_2_), arterial CO_2_ partial pressure (*P*aCO_2_), and arterial haemoglobin oxygen saturation (saO_2_).

For all blood gas analyses the same device (Radiometer ABL90) was used. This analyser has a validated range for *P*aO_2_ up to 550 mmHg. The measurements between 550 and 800 mmHg are not clinically validated and are therefore displayed as > 550 mmHg. Standardized and protocolized quality management procedures including calibration were performed daily according to the obligatory codes of action for laboratory devices of the German authorities [8].

Then, the first standardised preoxygenation manoeuvre was initiated. Over 5 min, 100% of oxygen was applied using a gas flow of 18 L per minute and a tight face mask. At the end of this manoeuvre, all measurements were repeated (M1). This was followed by breathing room air for 20 min to restore baseline (M2). If *P*aO_2_ at M2 was more than 10% higher than the respective value at M0, another 5 min of breathing room air was allowed and M2 was repeated. Then, the second standardised preoxygenation manoeuvre was initiated: Non-invasive ventilation (NIV) was implemented for a period of 5 min using 100% of oxygen, a tight fitted nose-mouth mask strapped to the patients’ head, a positive end-expiratory pressure of 5 mmHg and an assisted spontaneous breathing mode with a support pressure of 8 mmHg (Primus, Draeger, Lübeck, Germany). Data (M3) was taken after 5 min.

After this second preoxygenation the patient was intubated and mechanically ventilated. Two more sets of measurements were taken during the operation to observe the association between ORI and *P*aO_2_: One just before the first incision (M5) and one after 30 min of surgery (M7).

### Statistics

This study was designed as an explorative investigation on the new parameter ORI. Since only very limited data on ORI were available at the timepoint of the design, no conventional sample size calculation was performed. Descriptive statistics are presented as mean, standard deviation, median, 1st and 3rd quartile, minimum, maximum and number of observations for continuous variables and as absolute and percent frequencies for categorical variables. Data distributions of continuous variables were assessed by visual examinations of histograms and boxplots. Differences between M1 and M0 (M1_0_diff) and between M3 and M2 (M3_2_diff) were computed for ORI, *P*aO_2_ and SpO_2_. Paired t-tests were then used to determine whether M3_2_diff and M1_0_diff differed for these variables. Spearman rank correlation was employed to examine relationships between *P*aO_2_ and ORI. The first analysis considers all data points, in the second correlation analysis only the data points with a *P*aO_2_ of 100–200 mmHg were considered. Significance level α was 5%. All tests were two-tailed. We also tested how well the *P*aO_2_ threshold criteria suggested by Applegate et al. [9] (*P*aO_2_ ≥ 100 mmHg and *P*aO_2_ ≥ 150 mmHg, respectively) would predict ORI values exceeding 0.24 respectively 0.55 when applied to our data. Data with PaO_2_ values ≥ 550 mmHg were not included in the analyses to prevent the possible falsifying influence of the ceiling effect due to the limited measuring range. The resulting diagnostic key figures sensitivity, specificity, accuracy as well as positive and negative predictive value are reported. All statistical analyses were done using SPSS, version 27 (IBM Corp, Armonk, NY, USA).

## Results

From May 2017 until May 2018 41 patients were included in this study, 27 of them were female (65.9%). Patient data are given in Tables 1 and 2. In Table 3, hemodynamic data, data on pulse oximetry and ORI, as well as on blood gas analysis are given for M0–M3.

**Table 1:** Patient demographic data

N = 41	Mean ± SD	Min	Max
Age (years)	42.7 ± 10.9	18	66
Height (cm)	169.2 ± 8.9	143	186
Weight (kg)	150. 7 ± 27.8	105	240
BMI (kg/m^2^)	52.4 ± 7.7	40.4	72.5

*BMI* body mass index

**Table 2:** Patient diagnoses

Diabetes	24.4% (10)
Hypertension	53.7% (22)
OSAS	19.5% (8)
Asthma	19.5% (8)
Smoker	Active 17.1% (7) Quit < 5y ago 12.2% (5)

*OSAS* obstructive sleep apnoea syndrome

**Table 3:** Data on oxygenation and hemodynamics

Variable	MP	Mean	Median	Standard deviation	Minimum	Maximum	1^st^ quartile	3^rd^ quartile	Valid cases	Missing cases
ORI	0	0.01	0.00	0.06	0.00	0.40	0.00	0.00	41	0
	1	0.43	0.39	0.21	0.15	1.00	0.27	0.52	41	0
	2	0.00	0.00	0.00	0.00	0.00	0.00	0.00	41	0
	3	0.41	0.38	0.20	0.00	1.00	0.29	0.51	41	0
SpO_2_ (%)	0	97	97	2.2	92	100	96	99	41	0
	1	100	100	0.4	98	100	100	100	41	0
	2	96	97	2.2	91	100	95	98	41	0
	3	100	100	0.4	98	100	100	100	41	0
*P*aO_2_ (mmHg)	0	80.2	78.1	11.3	60.9	105.0	71.7	88.1	41	0
	1	486.0(#)	505.0(#)	74.4(#)	299.0	550.0(*)	447.0(#)	550.0(#)	41	0
	2	78.1	76.7	10.8	60.9	102.0	68.9	87.8	41	0
	3	543.8(#)	550.0(#)	17.6(#)	482.0	550.0(*)	550.0(#)	550.0(#)	41	0
*P*aCO_2_ (mmHg)	0	36.6	36.3	3.3	30.9	43.3	34.0	39.1	41	0
	1	36.6	36.6	4.2	29.7	45.2	32.8	40.0	41	0
	2	37.3	37.7	3.8	28.5	45.6	34.8	39.8	41	0
	3	32.9	32.9	5.1	20.7	45.7	29.6	36.1	41	0
MAP (mmHg)	0	101	100	13.5	74	134	91	107	41	0
	1	98	95	12.2	74	131	91	102	41	0
	2	100	96	12.5	81	129	93	109	41	0
	3	100	97	12.1	79	128	92	109	41	0
HR (1/min)	0	79	79	12.8	58	115	70	89	41	0
	1	76	73	12.4	59	108	67	85	41	0
	2	78	74	13.8	61	112	67	90	41	0
	3	76	75	13.9	54	108	66	86	41	0

(*): values were > 550 mmHg, blood gas analyser was limited to 550 mmHg

(#): influenced by ceiling effect due to the blood gas analyser being limited to 550 mmHg

*M* measuring point, *Std. deviation* standard deviation, *ORI* oxygen reserve index, *SpO*_*2*_ peripheral oxygen saturation, *PaO*_*2*_ partial arterial pressure of oxygen, *PaCO*_*2*_ partial arterial pressure of carbon dioxide, *MAP* mean arterial pressure, *HR* heart rate

### First baseline

At baseline (M0), patients presented with a median oxygen saturation of 97% (IQR 95.5–99%) and a *P*aO_2_ of 78.1 mmHg (IQR 71.7–88.1 mmHg). ORI was 0 (IQR 0–0). Two patients presented with an ORI > 0, although no additional oxygen was given (ORI 0.03 with a *P*aO_2_ 97.6 mmHg and ORI 0.4 with a *P*aO_2_ of 81.4 mmHg).

### First preoxygenation manoeuvre (standard preoxygenation)

After oxygenation with 100% oxygen for 5 min the median ORI level rose to 0.39 (IQR 0.27–0.52), values ranging from 0.15 to 1. In parallel, median SpO_2_ increased to 100% (IQR 100–100%). Median *P*aO_2_ values increased to 505 mmHg (IQR 447–550 mmHg). Twelve patients showed a *P*aO_2_ above 550 mmHg.

### Second baseline

After breathing room air for 20 min ORI values returned to zero and in all patients. In parallel, median SpO_2_ decreased to 97% (IQR 95–98%). *P*aO_2_ values decreased to a median value of 76.7 mmHg (IQR 68.9–87.8 mmHg). Only in one patient an additional time of 5 min was necessary to restore baseline. ORI dropped to zero in all patients.

### Second preoxygenation manoeuvre (NIV preoxygenation)

With the NIV preoxygenation the ORI increased to a mean value of 0.38 (IQR 0.28–0.51). Concomitantly, median SpO_2_ increased to 100% (IQR 100–100%), and *P*aO_2_ to a median of 550 mmHg (IQR 550–550 mmHg). At this point 36 of the 41 patients reached *P*aO_2_ values > 550 mmHg. Again, median and 1st and 3rd quartile for *P*aO_2_ were most likely underestimated.

### Assessment of effectiveness of preoxygenation by ORI

**Fig. 2:**
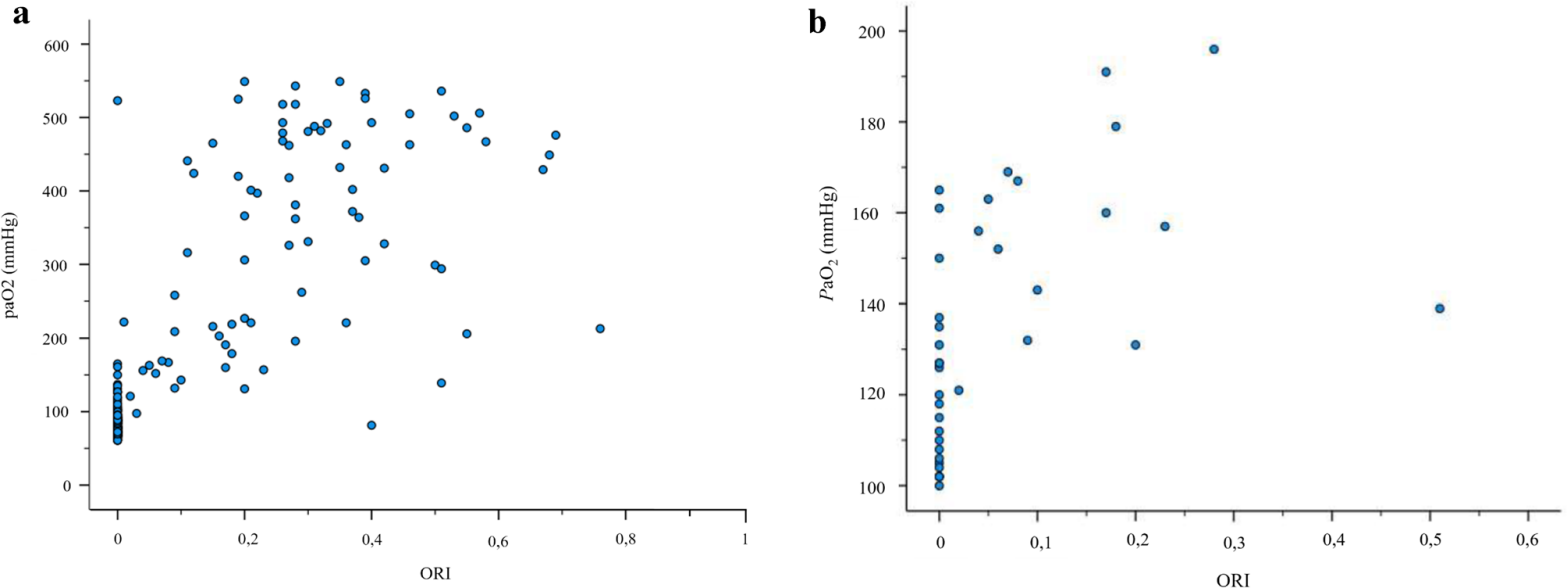
**a** Correlation Analysis Oxygen Reserve Index (ORI) vs. *P*aO_2_ (M 0–3, 5 and 7), datapoints for *P*aO_2_ ≥ 550 mmHg excluded. **b** Correlation Analysis Oxygen Reserve Index (ORI) vs. PaO2 (M 0–3, 5 and 7) for PaO2 100–200 mmHg. These scatterplots show all ORI values (x axis) with their corresponding *P*aO_2_ values (y axis)

Overall correlation of *P*aO_2_ and ORI: Fig. 2a illustrates a scatter plot of all *P*aO_2_-values and their respective ORI values (data from M 0–3, 5 and 7 without datapoints for *P*aO_2_-values ≥ 550 mmHg), showing a significant correlation between those two variables (Spearman-Rho coefficient of correlation 0.818, p < 0.001, n = 195). Figure 2b shows the correlation for *P*aO_2_ and ORI in the intended range for ORI of 100–200 mmHg *P*aO_2_ (Spearman-Rho coefficient of correlation 0.669, p < 0.001, n = 37).

**Fig. 3:**
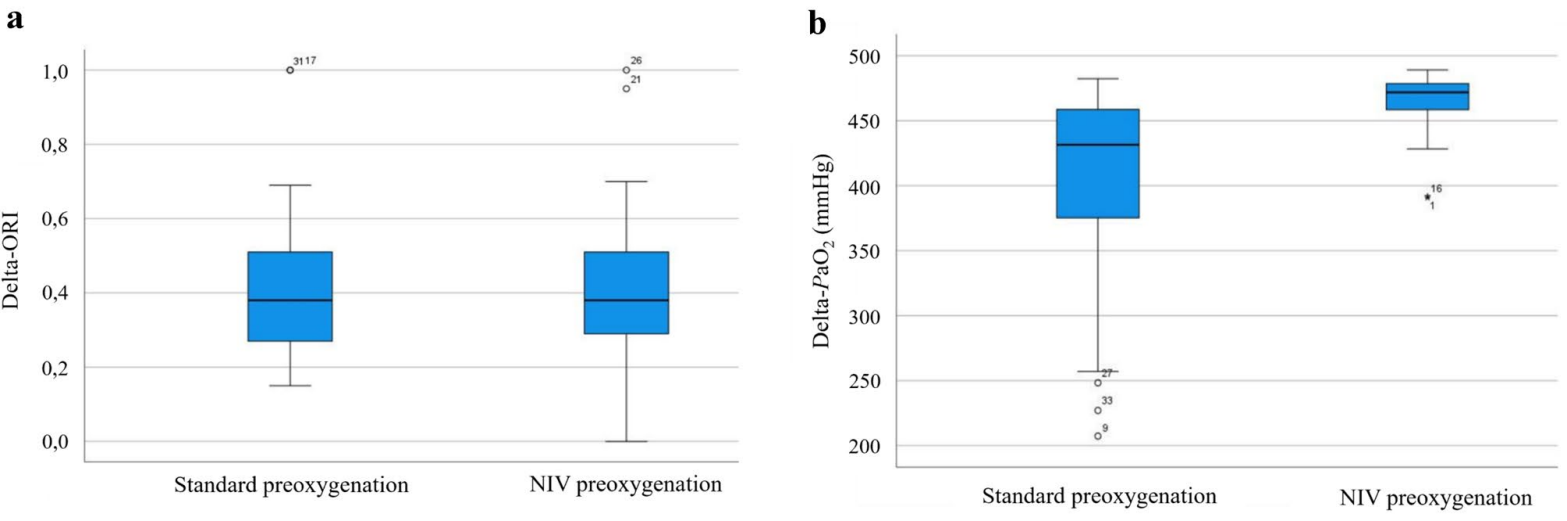
**a** Changes of ORI induced by preoxygenation manoeuvres. ORI, oxygen reserve index; M, measurement point. **b** Changes of *P*aO_2_ induced by preoxygenation manoeuvres. The boxplot in Fig. 3a shows the delta for ORI for the standard preoxygenation (M1–0) and NIV preoxygenation (M3–2) which shows no significant differences. The boxplot 3b shows the delta in *P*aO_2_ for standard preoxygenation (M1-M0) and NIV preoxygenation (M3–M2) with significant difference between the two maneuvers

Figure 3 illustrates the relation between changes in ORI (delta-ORI, Fig. 3a) and changes in *P*aO_2_ (delta- *P*aO_2_, Fig. 3b) induced by preoxygenation.

No association between the delta-ORI and the delta*-P*aO_2_, induced by the respective preoxygenation manoeuvres, was noticed.

**Fig. 4:**
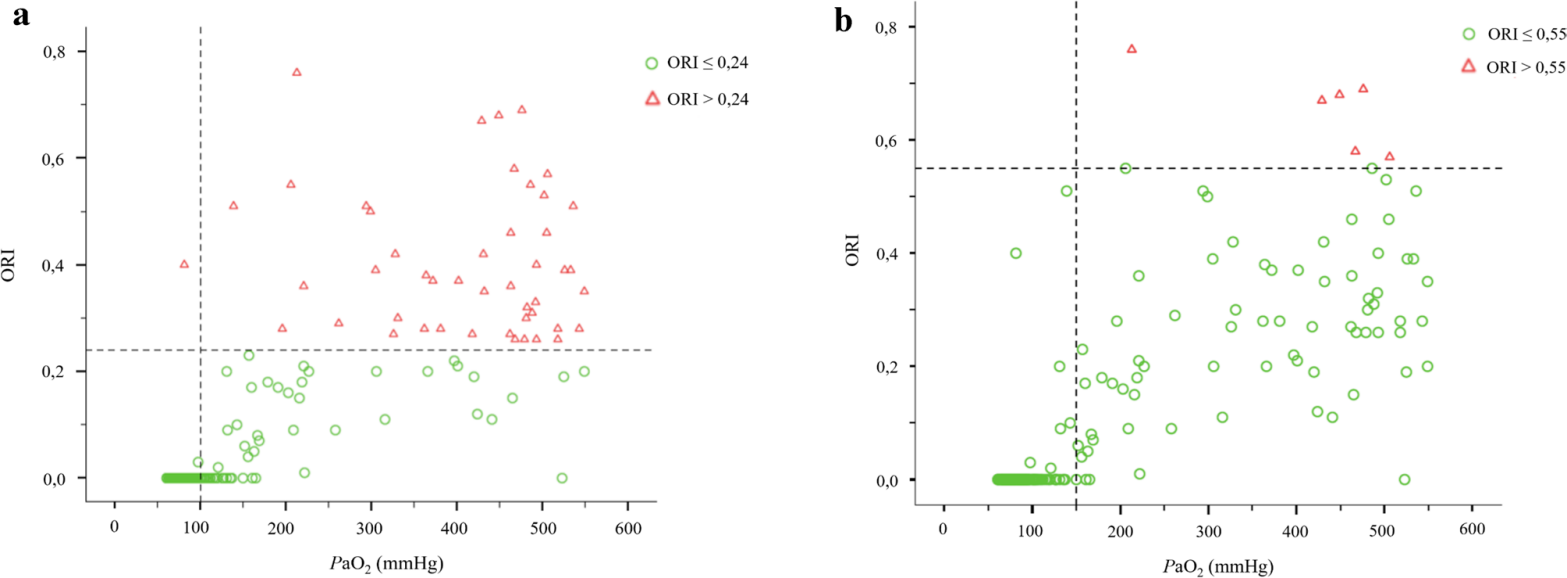
**a** Scatterplot for ORI > 0,24. **b** Scatterplot for ORI > 0,55. Red triangular data points in Fig. 4a and Fig. 4b indicate ORI values that exceeded the 0.24 (a) and 0.55 (b) thresholds. The corresponding horizontal threshold lines for ORI and vertical threshold lines for *P*aO_2_ ≥ 100 mmHg (**a**) and *P*aO_2_ ≥ 150 mmHg (**b**). Datapoints for *P*aO_2_ ≥ 550 mmHg excluded

Figure 4a und 4b show scatterplots of ORI with corresponding *P*aO_2_ values (data from M 0–3, 5 and 7 without datapoints for *P*aO_2_-values ≥ 550 mmHg). Red data points indicate ORI values exceeded the 0.24 and 0.55 thresholds, respectively, as suggested by Applegate et al. [9] The corresponding horizontal threshold lines for ORI and vertical threshold lines for *P*aO_2_ ≥ 100 mmHg and *P*aO_2_ ≥ 150 mmHg, respectively, are also shown. Applying these threshold values within a diagnostic test setting yields the following key figures for detecting ORI > 0.24 based on *P*aO_2_ ≥ 100 mmHg: accuracy = 71%, sensitivity = 98%, specificity = 63%, positive predictive value = 46% and negative predictive value = 99%. For detecting ORI > 0.55 based on *P*aO_2_ ≥ 150 mmHg, accuracy = 64%, sensitivity = 100%, specificity = 62%, positive predictive value = 8% and negative predictive value = 100%. Note that also a very low ORI can occur at high *P*aO_2_ values.

### Comparison of both preoxygenation manoeuvres and assessment with ORI

SpO_2_ values produced by both preoxygenation procedures did not differ significantly (median 100% for both procedures). *P*aO_2_ values reached by NIV preoxygenation were significantly higher compared standard preoxygenation (median 505 mmHg (M1) vs. 550 mmHg (M3); p < 0.0001) as depicted in Table 3. In contrast, ORI values did not differ significantly (median 0.39 (M1) vs. 0.38 (M3); p = 0.758).

NIV preoxygenation resulted in clinically significant higher *P*aO_2_ values compared to standard preoxygenation. We further analysed the respective increases in SpO_2_, *P*aO_2_ and ORI induced by standard preoxygenation and NIV preoxygenation: Standard preoxygenation resulted in a median increase in SpO_2_ of 3% (IQR 1–4%). The median increase in *P*aO_2_ was 431.6 mmHg (IQR 375.3–458.7 mmHg). NIV preoxygenation resulted also in an increase in SpO_2_ of 3% (IQR 2–5%). *P*aO_2_ increased by 471.9 mmHg (IQR 458.5–478.6 mmHg). The median increase in *P*aO_2_ was significantly higher in NIV preoxygenation vs. standard preoxygenation (p < 0.0001) and most likely was even higher due to the limitation to 550 mmHg of the measurement of *P*aO_2_. In contrast, the increase in SpO_2_ and ORI did not differ significantly between both procedures.

## Discussion

The main focus of this study was to assess, if ORI might be clinically useful to quantify the effect of preoxygenation on paO2 in a non-invasive way in morbidly obese patients. Our results do not support this hypothesis. Further, differences in the effectiveness of preoxygenation via facemask, or by NIV cannot be assessed by ORI.

Our data confirm that both preoxygenation techniques increase the *P*aO_2_ levels significantly in bariatric patients, and thus, increase the safety margin during airway management [2, 10]. We further could confirm that NIV helps to get an even better preoxygenation effect than the application of oxygen without pressure support [4, 11]. An interesting finding is that the *P*aO_2_ values reached with both preoxygenation techniques were considerably higher in our study compared to earlier investigations in obese patients [5, 11]. Why we achieved higher oxygen levels is up to speculation, since also the BMI matched the comparative studies. One explanation might be that in our study, all preoxygenation manoeuvres were performed by the same anaesthesiologist, so standardisation of the manoeuvres might have been higher. The high PaO2 values resulted in a ceiling effect especially for the NIV preoxygenation manoeuvre due to the blood gas analyser used in the standard setting. The measurements above 550 mmHg are imprecisely and are therefore reported as > 550 mmHg. We decided against the exclusion of the datapoints with PaO2 above 550 mmHg since that would have excluded almost all measurements of the NIV preoxygenation manoeuvre and the general trend is still assessable.

Preoxygenation with NIV resulted in higher *P*aO_2_ levels, thus, effectiveness of NIV preoxygenation was better. ORI levels after preoxygenation, however, did not differ between the standard preoxygenation and the NIV preoxygenation. Further, there was no association between changes in *P*aO_2_ and changes in ORI induced by preoxygenation. Thus, ORI did not allow to indicate this different effectiveness.

ORI uses the saturation of venous blood for its algorithm. However, at *P*aO_2_ values of approximately 200 to 250 mmHg the venous saturation reaches a plateau, and the ORI cannot measure any higher values [12, 13]. Recent studies gave evidence that the ORI can be useful in phases of mild hyperoxemia between 100 and 250 mmHg, and in particular in situations close to hypoxia [14, 15]. Since standard preoxygenation normally results in *P*aO_2_ levels above 200 mmHg, and therefore outside the range of sensitivity of ORI, any difference above could not be differentiated by this variable.

The reason we performed this study even though the range of ORI was described as being limited to an *P*aO_2_ between 100 and about 200 mmHg was that ORI was repeatedly suggested to be a new parameter to judge preoxygenation before induction of anaesthesia [13, 16]. Just recently, Hirata et al. reported on significant correlations between changes in ORI and concomitant changes in end-tidal oxygen during preoxygenation with a tight face mask in low-risk patients [16]. We agree that this gives additional information on the technical quality of preoxygenation manoeuvre, however it adds no information on the final physiological effect.

Applegate et al. [9] found a clustered relation between the magnitude of ORI and *P*aO_2_: If ORI was above 0.24 the *P*aO_2_ was supposed to be greater than 100 mmHg, and with an ORI of 0.55 the *P*aO_2_ was greater than 150 mmHg. Our data supports the conclusion that a certain ORI predicts a minimal *P*aO_2_ as shown in Fig. 4a and b but a reverse conclusion is not possible. And given that the intended range for ORI starts at 100 mmHg *P*aO_2_ the prediction of a *P*aO_2_ > 100 mmHg at an ORI of 0.24 seems irrelevant. Even very high *P*aO_2_ values were associated with low ORI values. This points out that the ORI and its changes under increases in *P*aO_2_ in its present form is inter-individually different. Thus, also estimation of patient individual achievable ORI values under preoxygenation seems not possible. Factors that influence the individual maximum might be all factors that influence peripheral perfusion but that should be subject of another study.

Limitations of this study: The blood gas analyser used in this study was restricted to measurements of *P*aO_2_ < 550 mmHg. Values above 550 mmHg were marked and set as 550 mmHg. This was the case in twelve single measurements at M1 and all but five times in M3. Time points where this was the case, are marked in Table 3 and were set to 550 mmHg for further calculations. This of course induced a bias to further calculations (median and IQR); those values need to be interpreted as underestimated.

In conclusion, the absolute values of ORI cannot be used to assess the effectiveness of a preoxygenation procedure in bariatric patients. This is mainly because its range of discrimination is considerably under the high ranges of *P*aO_2_ that are attained by adequate preoxygenation. ORI may be useful as an early indicator of impending return to normoxia from preoxygenation levels, which needs to be proven in future studies. Further, if in particular intra-individual changes of ORI over time might additionally help to characterise phases of deoxygenation should be further investigated.

## Data Availability

Not applicable.

## References

[1] Nimmagadda U, Salem MR, Crystal GJ (2017). Preoxygenation: physiologic basis, benefits, and potential risks. Anesth Analg.

[2] Bouroche G, Bourgain JL (2015). Preoxygenation and general anesthesia: a review. Minerva Anestesiol.

[3] Berthoud MC, Peacock JE, Reilly CS (1991). Effectiveness of preoxygenation in morbidly obese patients. Br J Anaesth.

[4] Carron M, Zarantonello F, Tellaroli P, Ori C (2016). Perioperative noninvasive ventilation in obese patients: a qualitative review and meta-analysis. Surg Obes Relat Dis.

[5] Delay JM (2008). The effectiveness of noninvasive positive pressure ventilation to enhance preoxygenation in morbidly obese patients: a randomized controlled study. Anesth Analg.

[6] Macintyre NR (2019). Physiologic effects of noninvasive ventilation. Respir Care.

[7] Cross M, Plunkett E (2008). Physics, pharmacology and physiology for anaesthetists.

[8] Schumann G (2018). Richtlinie der Bundesärztekammer zur Qualitätssicherung laboratoriumsmedizinischer Untersuchungen.

[9] Applegate RL, Dorotta IL, Wells B, Juma D, Applegate PM (2016). The relationship between oxygen reserve index and arterial partial pressure of oxygen during surgery. Anesth Analg.

[10] Jense HG, Dubin SA, Silverstein PI, O’Leary-Escolas U (1991). Effect of obesity on safe duration of apnea in anesthetized humans. Anesth Analg.

[11] Coussa M (2004). Prevention of atelectasis formation during the induction of general anesthesia in morbidly obese patients. Anesth Analg.

[12] “Whitepaper Oxygen Reserve Index ^TM^ (ORi ^TM^ ),” 2017.

[13] Scheeren TWL, Belda FJ, Perel A (2018). The oxygen reserve index (ORI): a new tool to monitor oxygen therapy. J Clin Monit Comput.

[14] Vos JJ (2019). Oxygen reserve index: validation of a new variable. Anesth Analg.

[15] Yoshida K (2020). Adjustment of oxygen reserve index (ORi^TM^) to avoid excessive hyperoxia during general anesthesia. J Clin Monit Comput.

[16] Hirata N, Nishimura M, Chaki T, Yoshikawa Y, Yamakage M (2021). Comparison between oxygen reserve index and end-tidal oxygen concentration for estimation of oxygenation during pre-oxygenation via a tight-fitted face mask. Eur J Anaesthesiol.

